# All-Year High IAA and ABA Contents in Rhizome Buds May Contribute to Natural Four-Season Shooting in Woody Bamboo *Cephalostachyum pingbianense*

**DOI:** 10.3390/plants13030410

**Published:** 2024-01-30

**Authors:** Wei Mao, Changyan Bao, Qian Cheng, Ning Liang, Lianchun Wang, Hanqi Yang

**Affiliations:** 1 Faculty of Foreign Languages, Southwest Forestry University, Kunming 650233, China; mwei99@sina.com; 2 Institute of Highland Forest Science, Chinese Academy of Forestry, Kunming 650233, China; baochangyan@caf.ac.cn (C.B.); cqq08042@163.com (Q.C.); ln417@126.com (N.L.); 3 Key Laboratory of Breeding and Utilization of Resource Insects, National Forestry and Grassland Administration, Kunming 650233, China; 4 Forestry College, Southwest Forestry University, Kunming 650233, China

**Keywords:** *Cephalostachyum pingbianense*, bamboo shoot, endogenous hormones, ABA, IAA, rhizome bud

## Abstract

To explore the regulation mechanism of endogenous phytohormones on rhizome bud germination in *Cephalostachyum pingbianense*, the contents of IAA, ABA, GA, and CTK in seven above- and under-ground bamboo structure components were determined using enzyme-linked immunosorbent assays (ELISA). The results showed that a higher content of IAA, GA, and CTK all year was found in above-ground components and dormant rhizome buds. Meanwhile, a higher ABA content in young shoots and a lower ABA content in the culm base and dormant rhizome buds were detected during the peak period of shooting. The amounts of emerging shoots and the grown bamboo culms were positively correlated with the content of IAA and the ratio of IAA/ABA and (IAA + CTK + GA)/ABA, while they were negatively correlated with the ratio of CTK/IAA in dormant rhizome buds. The all-year high contents of IAA (19–31 ng/g) and ABA (114–144 ng/g) in rhizome buds, as well as interactions among four hormones, may be the key physiological mechanisms to maintain rhizome bud germination throughout the year in *C. pingbianense*. As *C. pingbianense* is a special bamboo species of multi-season shoot sprouting, the above results may supplement scientific data for a comprehensive understanding of physiological mechanisms within the bamboo subfamily.

## 1. Introduction

Endogenous phytohormones are compounds synthesized by plants in trace amounts that participate in regulating the entire growth process of plants and stress responses to the natural environment. Therefore, endogenous phytohormones play a key role in the life span of plants [1,2,3,4,5]. Tiller from the lateral bud is an important form of plant growth, especially in clonal plants. Various hormones, such as auxin/IAA, cytokinin (CTK), gibberellin (GA), and abscisic acid (ABA), are involved in the process of lateral bud germination [1,5]. For example, ABA can affect the expression of key transcription factors that maintain bud dormancy [6,7], while IAA [8,9,10], CTK [11,12], and GA [13] cooperatively promote germination and development of lateral buds in multiple plants.

The rhizome bud germination of bamboo, i.e., bamboo shooting, is the main pathway of asexual reproduction in woody bamboos, as well as the foundation of bamboo resource cultivation and bamboo industry [14,15,16]. According to the type of underground rhizome, woody bamboos can be totally divided into two types: the scattered bamboos and the clump bamboos, which display different shooting phenologies. The scattered bamboo species (such as *Phyllostachys edulis*) are distributed in the subtropical zone and typically experience a shooting peak from March to May. On the other hand, the clump bamboos occur in tropical regions (such as *Dendrocalamus latiflorus*) and have a shoot-sprouting period from June to August. Both types start shooting soon after the beginning of the rainy season [15,16]. Therefore, empirically, the rhizome buds germination of woody bamboos with a single shooting peak is generally considered to be induced by the regulation of endogenous hormone synthesis and transport following an increase in soil temperature and precipitation in the rainy season [8]. The previous studies indicated the mass fractions of various phytohormones fluctuated regularly during the shooting period, implying that antagonism or coordination of endogenous hormones, such as IAA, GA, and ABA, were key signaling factors for bamboo shoot initiation [17,18,19].

Woody bamboos are typical clonal plants, and endogenous hormones will transport and regulate physiological activity in the whole gene system [20]. In the present study, we adopted the hypothesis that there is extensive in situ synthesis and transport of endogenous hormones among different ramets (or culms) of bamboo genet, and these phytohormones synergistically regulate the germination of rhizome buds and culm growth and development [14]. Although abundant achievements have been made in the mechanism of bamboo rhizome bud germination, there are still some defects in these studies: (1) mostly focused on bamboo species with a single sprouting peak, especially scattered bamboo; (2) Previous studies, as far as we knew, were only based on the shooting season (lasting ca. 3–4 months), without examining the content changes of endogenous hormones throughout the year, e.g., [17,18,19]; (3) Most studies only focused on rhizome and its lateral buds and disregarded the connection between the above-ground and underground components of bamboo genet, which may be due to the large and complex culm branching and rhizome system of woody bamboos [21]. Thus far, the precise regulatory mechanism of endogenous hormones on rhizome bud germination in bamboo is still largely unclear.

Compared to the infinitely extended rhizome system in the scattered bamboos, the clone ramets of the clump bamboos are clustered together, similar to rice, forming an ideal and closed observation system for in vivo endogenous phytohormones, which is very conducive to studying the biosynthesis and transmission process of endogenous hormones among clone ramets in bamboos. *Cephalostachyum pingbianense* is a small-scaled cluster bamboo and mainly occurs in southern Yunnan, China [22]. *C. pingbianense* is currently the only known bamboo species that can produce bamboo shoots throughout the year under natural conditions. According to existing research, this species has two peak periods for shoot sprouting, namely in autumn and spring [23,24]. Compared with the majority of woody bamboos with a single shooting peak, *C. pingbianense* displays an interesting shooting phenology and provides an ideal experimental material for studying the germination mechanism of bamboos, which is of great significance for understanding the mechanism diversity of rhizome buds germination within woody bamboos.

In this article, we fixed-site observed shooting situations, examined the concentration changes of four key endogenous hormones, i.e., IAA, CTK, GA, and ABA, in the above-ground and underground components of the entire ramet system during four seasons in *C. pingbianense* clumps, and further analyzed the correlation between endogenous hormones and the amounts of the emerging and degraded shoots in each season. Thus, we inferred the possible regulatory mechanism of endogenous hormones on naturally occurring four-season shooting in *C. pingbianense*. This study is helpful to the understanding of the biosynthesis and interactions of endogenous hormones in the ramets of *C. pingbianense*, which provides a reference for analyzing its unique shooting mechanism and improves our comprehensive understanding of the diversity of shooting mechanisms in woody bamboos.

## 2. Results

### 2.1. Sprouting Phenology and Quantity of Bamboo Shoots

**Figure 1:**
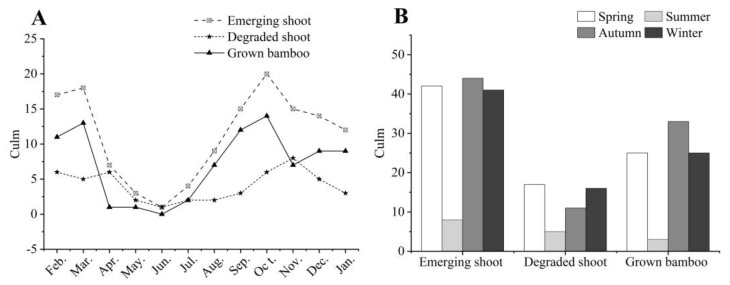
Emergent and degraded bamboo shoot amounts in six sample clumps of *C. pingbianense*. (**A**) Monthly and (**B**) seasonal bamboo shoot amounts.

The phenology observation results of shoot sprouting showed that *C. pingbianense* could produce bamboo shoots throughout the year (Table 1, Figure 1). The least number of shoots appeared in June, with only one shoot out of six clumps. For the convenience of discussion, we assumed that June is the initial stage of the bamboo shooting of *C. pingbianense*. Meanwhile, *C. pingbianense* displayed two peak periods of shooting, with the highest shoot number in October, followed by March (Figure 1A). According to the season division, the highest number of shoots appeared in the autumn, followed by the spring. The season with the highest number of degraded shoots was spring, followed by winter (Figure 1B). The highest survived shoot number was in autumn, making up 38.3% of the total number of survived bamboo shoots, while the lowest was in summer (3.5%). The number of bamboo shoots that survived in the spring was almost equal to that in the winter. Overall, the peak period of degraded bamboo shoots was delayed by about one month compared to the peak period of emergent shoots, and the number of survived bamboo shoots (or young bamboo) was positively correlated with the number of sprouting shoots.

### 2.2. Annual Spatial-Temporal Distribution of Endogenous Hormones

**Figure 2:**
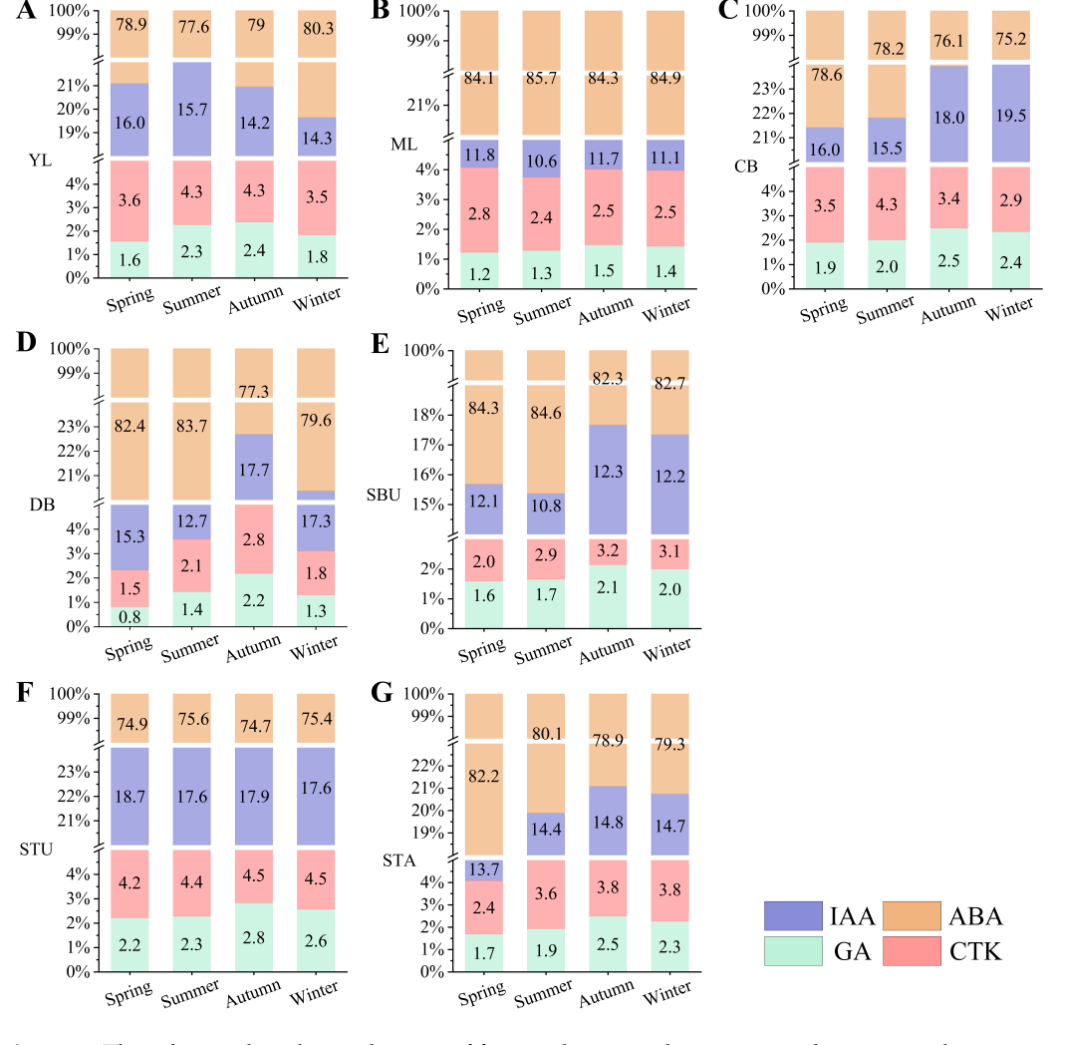
The relative abundance changes of four endogenous hormones in the examined components of *C. pingbianense*. (**A**) Young leaves (YL), (**B**) mature leaves (ML), (**C**) culm base (CB), (**D**) dormant rhizome buds (DB), (**E**) shoot base underground (SBU), (**F**) shoot tips underground (STU), and (**G**) shoot tips above-ground (STA).

The results of content determination with the fresh weight of four endogenous hormones in *C. pingbianense* indicated that the mass fraction of four hormones in different components was significantly different (Figure 2). In each examined component, ABA had the highest content of 89–144 ng/g, with a relative abundance of 74.7–85.7%. The following sequence of content was as follows: IAA 15–31 ng/g (10.6–19.5%), CTK 2.6–6.7 ng/g (1.5–4.5%), and GA 1.4–4.0 ng/g (0.8–2.5%).

**Figure 3:**
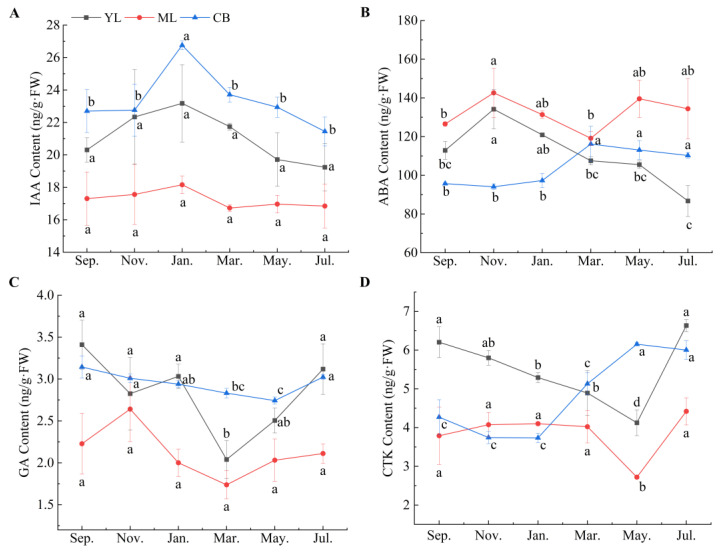
The annual content changes of four endogenous hormones in the above-ground components. Different lowercase letters in each sub-figure indicate significant differences at *p* < 0.05. (**A**) IAA content; (**B**) ABA content; (**C**) GA content; (**D**) CTK content. YL, young leaves; ML, mature leaves; CB, culm base.

**Figure 4:**
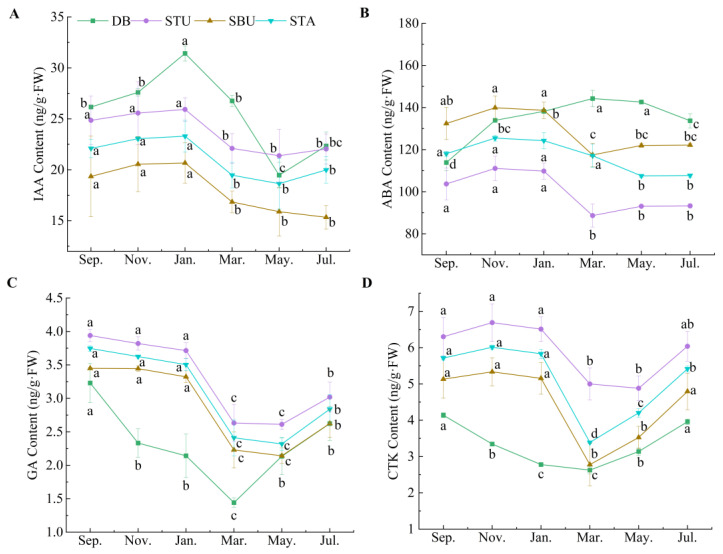
The annual content changes of four endogenous hormones in the underground components. Different lowercase letters in each sub-figure indicate significant differences at *p* < 0.05. (**A**) IAA content; (**B**) ABA content; (**C**) GA content; (**D**) CTK content. DB, dormant rhizome buds; STU, shoot tip underground; SBU, shoot base underground; STA, shoot tip above-ground.

The annual content changes of four endogenous hormones in seven components of *C. pingbianense* were as follows (Figure 3 and Figure 4).

IAA: Spatially, the contents of IAA in the underground components (including dormant rhizome buds and shoots) were totally higher than those of the above-ground components (including young leaves, mature leaves, and culm bases), and young tissues such as young leaves and shoot tips had much higher IAA contents than mature tissues such as culm bases and mature leaves. Temporally, IAA contents in the examined components were totally higher in the peak period of shoot sprouting, such as spring, autumn, and winter, than in the initial stage, i.e., from May to July.

ABA: Spatially, the highest average content of ABA was in dormant rhizome buds, and the lowest one was in culm bases. Totally, dormant rhizome buds and mature tissues such as mature leaves and shoot bases underground had a higher average ABA content than shoots, especially in shoot tips. Temporally, among the above-ground components, both young leaves and mature leaves had the highest ABA content in November, while the lowest contents occurred in March in mature leaves and in July in young leaves. Culm bases displayed an opposite trend, namely, the highest value was in March and the lowest value was in November. On the other hand, among the underground components, the shoots had the lowest ABA content in March or May and the highest content in November. Conversely, dormant rhizome buds had the highest ABA content in March and the lowest value in November.

GA: Spatially, dormant rhizome buds had the lowest average GA contents among the examined components, and as a whole, the underground components had a higher GA content than the above-ground components. Among the three above-ground components, mature leaves had the lowest GA contents. Temporally, in the peak period of shoot sprouting around September, the highest GA contents in the examined components were observed, while the lowest contents were found in the initial stage around May. Among them, the lowest GA contents in young leaves, mature leaves, and dormant rhizome buds appeared in March.

CTK: Spatially, taken overall, the CTK contents of underground components were higher than in the above-ground components, and the CTK contents of young tissues such as young leaves and shoot tips were much higher than those in mature tissues. Similar to GA, dormant rhizome buds also had the lowest average CTK contents among the examined components. Temporally, both young leaves and mature leaves had the lowest CTK content in May and the highest value in July, while culm base had the highest CTK content in May and the lowest CTK value in November. Among the underground components, shoots and rhizome buds had the lowest CTK contents around March and the highest contents around November.

### 2.3. Correlation Analysis of Endogenous Hormones 

#### 2.3.1. Endogenous Hormone Content Distribution at the Component Level

**Figure 5:**
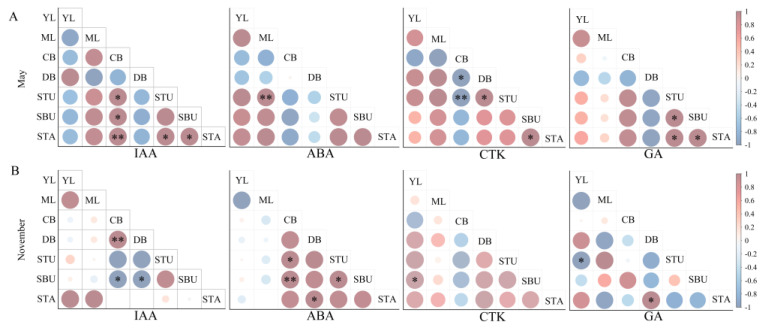
Pearson correlation analysis of a single endogenous hormone at different components. * indicates *p* < 0.05, and ** indicates *p* < 0.01. (**A**) Initial period of shooting in May; (**B**) peak period of shooting in November. YL, young leaves; ML, mature leaves; CB, culm base; DB, dormant rhizome buds; SBU, shoot base underground; STU, shoot tip underground; STA, shoot tip above-ground. The size and shade of the disc were directly proportional to the absolute value of the correlation coefficient.

To explore possible biosynthesis and transportation of the examined endogenous hormones among different components, we selected the data of endogenous hormone contents at the initial period (May) and peak period (November) of shooting phenology to analyze the distribution correlation of endogenous hormones at the component level. The correlation analysis results were as follows (Figure 5).

Initial period (May): IAA contents in culm base (CB) were positively correlated with those in shoot tip underground (STU) and shoot base underground (SBU), respectively, and had a significantly positive correlation with that in shoot tip above-ground (STA) (*p* < 0.01). Meanwhile, the IAA content in STA was positively correlated with that in STU and SBU, respectively. As for ABA content, only one significantly positive correlation was detected between mature leaves (ML) and STU. In terms of CTK content, CB was negatively correlated with dormant rhizome buds (DB) (*p* < 0.05) and STU (*p* < 0.01). Moreover, a positive correlation was found between DB and STU, as well as between SBU and STA, respectively. In terms of GA content, STU was positively correlated with SBU and STA, respectively, and a positive correlation existed between SBU and STA (Figure 5A).

Peak period (November): IAA content in CB was significantly positively correlated with DB, while IAA content in SBU was negatively correlated with CB and DB, respectively. In terms of ABA content, CB was positively correlated with STU (*p* < 0.05) and SBU (*p* < 0.01), respectively. In addition, DB was positively correlated with STA, and STU was also positively correlated with SBU. Regarding CTK content, young leaves (YL) were positively correlated with SBU. As for GA content, YL was negatively correlated with STU, and DB was positively correlated with STA (Figure 5B).

#### 2.3.2. Endogenous Hormone Interactions at the Bamboo Clump Level

**Figure 6:**
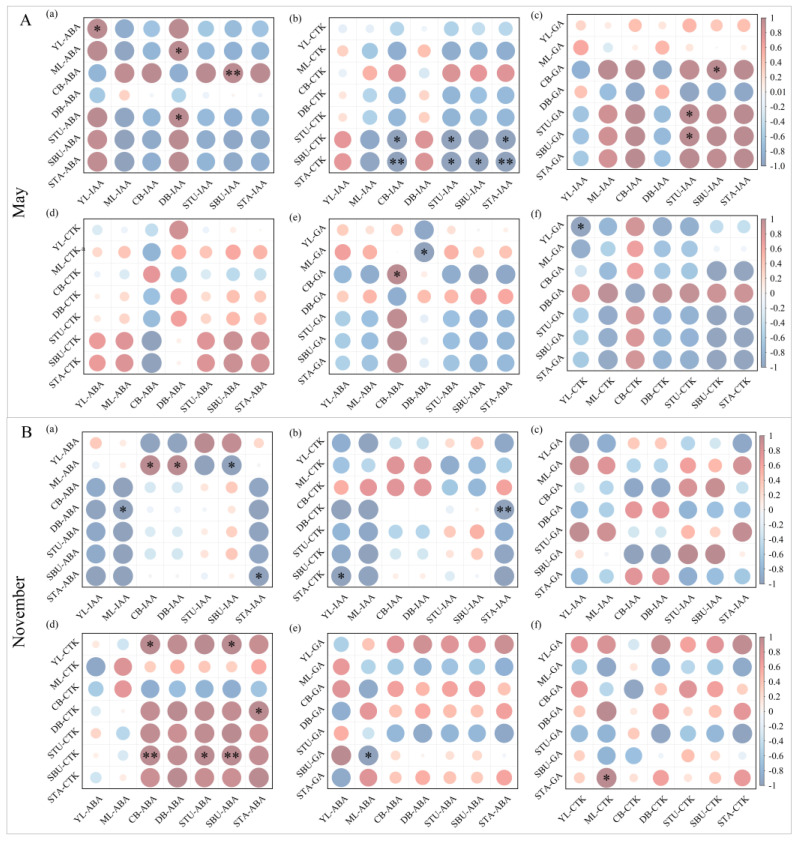
Pearson correlation analysis of four endogenous hormones in seven components. * indicates *p* < 0.05, and ** indicates *p* < 0.01. (**A**) Initial period of shooting in May; (**B**) peak period of shooting in November. In both sub-figures (**A**) and (**B**), (a) correlation between ABA and IAA; (b) correlation between CTK and IAA; (c) correlation between GA and IAA; (d) correlation between CTK and ABA; (e) correlation between GA and ABA; (f) correlation between GA and CTK. YL, young leaves; ML, mature leaves; CB, culm base; DB, dormant rhizome buds; SBU, shoot base underground; STU, shoot tip underground; STA, shoot tip above-ground. The size and shade of the disc were directly proportional to the absolute value of the correlation coefficient.

To investigate interactions among the examined endogenous hormones, the correlations among four hormones at the initial period (May) and peak period (November) were analyzed at the bamboo clump or genet level. Results showed that correlations between different hormones were significantly different in seven components at two typical periods of shooting phenology (Figure 6). 

Initial period (May): IAA had some specific correlations with ABA, CTK, and GA in different components (Figure 6A). As shown in Figure 6A(a), four positive correlations between IAA and ABA were detected, namely both IAA and ABA in YL (young leaves); IAA in DB (dormant rhizome buds) vs. ABA in ML (mature leaves) and STU (shoot tips underground); and IAA in SBU (shoot base underground) vs. ABA in CB (culm base). On the other hand, there were seven negative correlations between IAA and CTK, including IAA in CB vs. CTK in STA (shoot tip above-ground) and SBU; IAA in STU vs. CTK in STA and SBU; IAA in SBU vs. CTK in STA; and IAA in STA vs. CTK in SBU and STA. In addition, three positive correlations between IAA and GA were found, namely IAA in STU vs. GA in STU and SBU; and IAA in SBU vs. GA in CB. No significant correlation was detected between CTK and ABA. Two significant correlations were detected between GA and ABA, i.e., ABA and GA in CB; and ABA in DB vs. GA in ML. Only one significant correlation was found between CTK and GA, i.e., CTK content was negatively correlated with GA in YL (Figure 6A). The above results might imply that IAA had a synergistic effect with ABA and GA in the corresponding components, while an antagonistic effect may exist between IAA and CTK. 

Peak period (November): Five significant correlations were detected between IAA and ABA, including two positive (IAA in CB vs. ABA in ML; IAA in DB vs. ABA in ML) and three negatives (IAA in ML vs. ABA in DB; IAA in SBU vs. ABA in ML; IAA in STA vs. ABA in STA). Two negative correlations were found between IAA and CTK, i.e., IAA in YL vs. CTK in STA; and IAA in STA vs. CTK in DB. No significant correlation was detected between IAA and GA. Six positive correlations, including ABA in CB vs. CTK in YL and SBU; ABA in STU vs. CTK in SBU; ABA in SBU vs. CTK in YL and SBU; and ABA in STA vs. CTK in DB, were detected between ABA and CTK, indicating ABA might have a strongly synergistic effect with CTK. Only one negative correlation was found between ABA and GA, i.e., ABA in ML vs. GA in SBU. Meanwhile, only one positive correlation was found between CTK and GA, i.e., CTK in ML vs. GA in STA (Figure 6B).

### 2.4. Correlation Analysis between Bamboo Shoot Quantity and Endogenous Hormone Contents 

**Figure 7:**
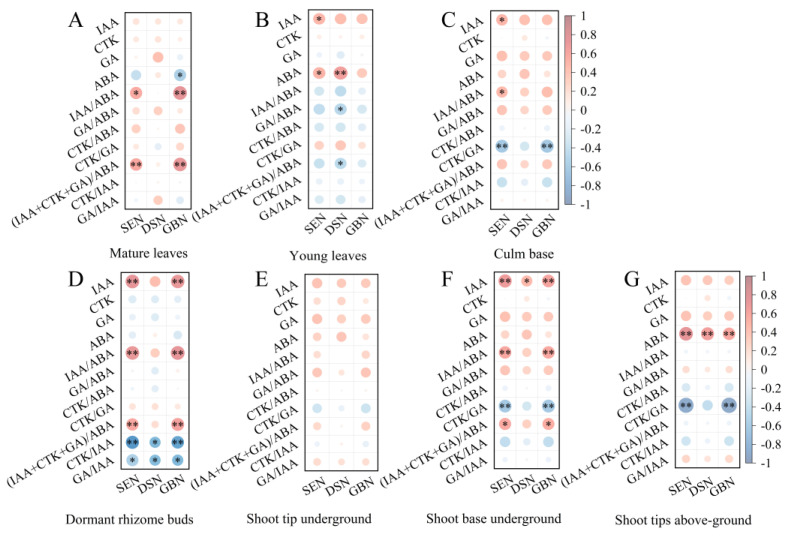
The correlation matrix between the number of bamboo shoots with different growth stages and endogenous hormone indicators from seven components. SEN: shoot emergency number; DSN: degraded shoot number; GBN: growing bamboo number. * indicates *p* < 0.05, and ** indicates *p* < 0.01. (**A**) Mature leaves; (**B**) young leaves; (**C**) base of bamboo culm; (**D**) dormant rhizome buds; (**E**) shoot tip underground; (**F**) shoot base underground; (**G**) shoot tip above-ground. The size and shade of the disc were directly proportional to the absolute value of the correlation coefficient.

The quantity of bamboo shoots is one of the most important goals in bamboo forest cultivation and management. In order to explore the impact of hormone interactions on the phenology of shoot sprouting, we analyzed the correlations between 11 kinds of possible hormone interactions in seven various components: shoot emergency number (SEN), degraded shoot number (DSN), and growing or survived bamboo culm number (GBN) (Figure 7).

The results showed that there were significant differences in the correlation between hormone indicators and SEN, DSN, and GBN at the component level (Figure 7). In the aspect of SEN, six positive correlations (including IAA/ABA in ML; IAA and ABA in YL; IAA and IAA/ABA in CB; (IAA + CTK + GA)/ABA in SBU, with *p* < 0.05 respectively) and seven significantly positive correlations (including (IAA + CTK + GA)/ABA in ML; IAA, IAA/ABA, and (IAA + CTK + GA)/ABA in DB; IAA and IAA/ABA in SBU; and ABA in STA, with *p* < 0.01 respectively) were found. At the same time, one negative correlation (namely GA/IAA in DB) and four significantly negative correlations (including CTK/GA in CB; CTK/IAA in DB; CTK/GA in SBU; and CTK/GA in STA) were detected.

In terms of DSN, one positive correlation (IAA in SBU) and two significantly positive correlations (ABA in YL and ABA in STA) were detected, while four negative correlations (i.e., GA/ABA and (IAA + CTK + GA)/ABA in YL; CTK/IAA and GA/IAA in DB) were found.

As for GBN, one positive correlation ((IAA + CTK + GA)/ABA in SBU) and eight significantly positive correlations (including IAA/ABA and (IAA + CTK + GA)/ABA in ML; IAA, IAA/ABA and (IAA + CTK + GA)/ABA in DB; IAA and IAA/ABA in SBU; ABA in STA) were detected, as well as two negative correlations (ABA in ML and GA/IAA in DB) and four significantly negative correlations (including CTK/GA in CB; CTK/IAA in DB; CTK/GA in SBU; and CTK/GA in STA).

According to correlation quantity, dormant rhizome buds (DB, 12 correlations including eight significant correlations) were the most closely related to the shoot sprouting phenology of *C. pingbianense*, followed by shoot base underground (SBU, nine correlations including six significant correlations), shoot tips above-ground (STA, five correlations and all were significant correlations), mature leaves (ML, five correlations including three significant correlations), young leaves (YL, five correlations including one significant correlation), culm base (CB, four correlations including two significant correlations), and shoot tips underground (STU, 0 correlation). Therefore, DB was the closest component related to the shooting phenology of *C. pingbianense*, a situation that may largely reflect the differences in the role of various components in the biological process of bamboo shoot sprouting.

## 3. Discussion

### 3.1. Annual Spatio-Temporal Distribution Changes of Endogenous Hormones

The ecological adaptation, growth, and development of plants, including woody bamboo, are influenced by both biological characteristics and the natural environment [25,26,27]. Among biological characteristics, endogenous hormone regulation is one of the main regulatory mechanisms for clonal reproduction and rapid growth of woody bamboo [28,29,30]. Due to the vast clonal lineage and the complex above-ground and underground branching systems of woody bamboo, the physiological regulation mechanism of bamboo shooting is greatly hindered. The previous studies have mostly studied content changes in endogenous hormones over a limited time span (usually a shooting period of 3–6 months) and limited components (mainly rhizomes, shoot buds, and above-ground branching), e.g., [16,17,18]. The peculiar biological characteristic of producing shoots every month in *C. pingbianense* provides valuable experimental materials to explore the physiological mechanism of bamboo shooting [23,24,31]. At the same time, the present study was based on the overall perspective of cloned plants. It measured endogenous hormone changes in the main above-ground and underground components of the entire bamboo genet throughout four seasons, which could more accurately grasp the changing trends of endogenous hormones in various components and provide comprehensive data for hormone biosynthesis.

Empirically, under natural conditions, most woody bamboos can produce shoots during a rhizome bud sprouting period of two to four months, which spans over a period of a season such as spring (mostly popular in the scattered bamboos) or summer (popular in the clump bamboos) [15,21]. Compared with the conventional single-season shooting bamboos, such as the scattered bamboos *Phyllostachys praecox* [17], *Ph. edulis* [19], and the clumps bamboo *Dendrocalamus latiflorus* [30], the contents of the four endogenous hormones in *C. pingbianense* were maintained at a relatively high level throughout the year (Figure 2, Figure 3 and Figure 4), namely ABA 89–144 ng/g, IAA 15–31 ng/g, GA 1.4–4.0 ng/g, and CTK 2.6–6.7 ng/g. Moreover, all orders of hormone content at seven components in the four seasons were consistent, i.e., ABA > IAA > CTK > GA. In particular, *C. pingbianense* possessed significantly high contents of ABA and IAA throughout the year, which was significantly different from the reported bamboo species, e.g., [17,18,19]. This may be one of the internal physiological regulatory mechanisms of the four-season shooting in *C. pingbianense* under natural conditions [16].

Moreover, there were significant differences in hormone content among the four components. The contents of IAA, CTK, and GA were higher in the meristem than those in mature tissues, respectively, while ABA content was higher in mature tissues than those in the meristem. It may offer new clues to explore the possible biosynthesis of the examined endogenous hormones among different components; for example, IAA may be synthesized in situ in meristems such as the shoot tips and young leaf tips [20,32,33]. Temporally, four hormone contents changed, apparently at the component level. The examined components had higher IAA, CTK, GA (in dormant buds), and ABA (in meristems) contents at the peak period of shooting than those at the initial stage. However, the ABA contents in the culm base and dormant buds during the initial period of shooting were higher than those during the shooting peak period. It may be related to the physiological activity of ABA in promoting embryonic tissue growth and early seedling development [34,35]. Moreover, the increased amount of ABA in shoot tips above-ground (STA) and shoot tips underground (STU) (Figure 4B) from the initial period (rainy season) to the peak period (dry season) of bamboo shooting may be because of the accumulation of ABA induced by drought in plants [36,37].

### 3.2. Correlation between Bamboo Shooting Phenology and Endogenous Hormones

The shoot sprouting period in common bamboo often lasts for approximately three months, and bamboo shoot production is often associated with an increase in precipitation and soil temperature [14,16,21]. However, the results of this study indicated that there was not a strong correlation between the shooting phenology of *C. pingbianense* and local natural hydrothermal conditions [24]. For instance, despite the relatively low soil temperature in winter, the shoot quantity reaches its peak. Meanwhile, the summer, with the best hydrothermal conditions, is actually the period of a year with the lowest shoot number. This may reflect the unique biological characteristics of *C. pingbianense* [22]. At the same time, this article verified that shoot sprouting throughout the year was the biological characteristic of *C. pingbianense*. Furthermore, the number of shoots fluctuated from season to season, mainly in relation to spring and summer [23,24]. Therefore, the shoot yield of *C. pingbianense* forest showed a fluctuating trend under natural conditions.

The results of this article further reveal that the three components most closely related to the shot phenology were the dormant rhizome bud, shoot base underground, and shoot tips above-ground. Dormant rhizome buds are the precursor of bamboo shoots, which can develop into shoots underground after germination. During the sprouting process of the rhizome bud, the biosynthesis or transport of endogenous hormones plays an important regulatory role [19,28]. Our result also exhibited that the amounts of shoot and young bamboo culm were positively correlated with IAA content, ratio of IAA/ABA, and (IAA + CTK + GA)/ABA, but were negatively correlated with ratio of CTK/IAA and GA/IAA in dormant rhizome buds. These results indicated that all four endogenous hormones were involved in the physiological regulation processes of rhizome bud germination and young bamboo culm formation. In addition, the above result also confirmed that in the regulation process of breaking shoot dormancy, an appropriate higher mass fraction of IAA may promote shoot sprouting, while higher contents of ABA, CTK, and GA have an inhibitory effect on shoot sprouting [17,19]. Furthermore, it was worth noting that the number of degraded shoots was significantly and positively correlated with ABA contents in young leaves (YL) (Figure 7B) and shoot tip above-ground (STA) (Figure 7G). This correlation was probably related to the function of ABA in promoting carbohydrate transportation and metabolism among components [18,38,39]. In summary, the endogenous hormones in bamboo components often exhibit synergistic or antagonistic interactions, thereby regulating rhizome bud germination and nutrient allocation between components in *C. pingbianense*, leading to phenological phenomena such as shoot sprouting and degraded shoots [16,19,40,41].

## 4. Materials and Methods

### 4.1. Plant Material

The sample collection site is located in the central distribution area of *C. pingbianense* in the Daweishan National Nature Reserve in southeastern Yunnan Province (22°54′ N, 103°42′ E). From September 2020 to September 2021, six thriving bamboo clumps (30–40 culms per clump) were randomly selected for the fixed observation and sampling of bamboo clumps. The numbers of sprouting and degraded shoots were recorded once a month, and endogenous hormone samples were collected every two months.

**Figure 8:**
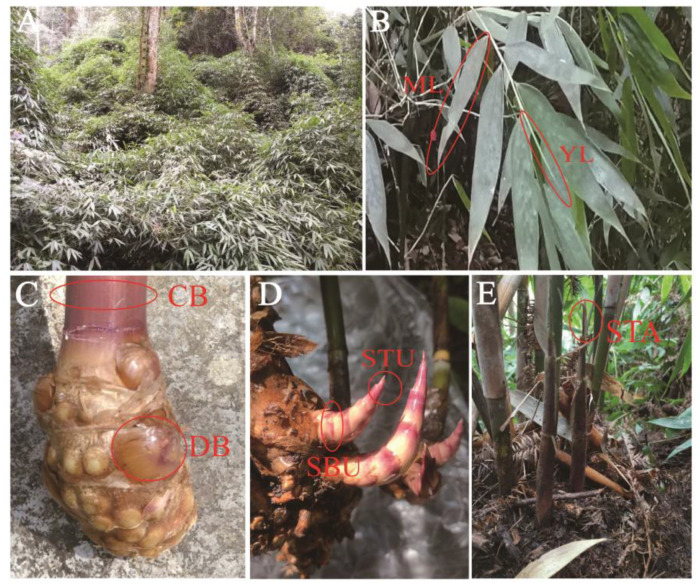
Habitat and structural components examined in C. pingbianense. (**A**) Habitat; (**B**) young leaves (YL), mature leaves (ML); (**C**) culm base (CB), dormant rhizome buds (DB); (**D**) shoot base underground (SBU), shoot tip underground (STU); (**E**) shoot tip above-ground (STA).

The components for collecting endogenous hormone samples were as follows: (1) young leaves (YL) at the top of the branches; (2) mature leaves (ML) at the lower part of the branches; (3) basal internodes of the adult bamboo culm, namely, culm base (CB); (4) dormant rhizome buds (DB); (5) shoot base underground (SBU); (6) shoot tips underground (STU); (7) shoot tips above-ground (STA), approximately 10–15 cm shoot tips unearthed (Figure 8). Following conventional or empirical views, we regarded each clump as a potential genet and the culms within as ramets of a clone in clump or sympodial bamboos [14,15,21]. The sampling components in this study represented the developmental stages of bamboo culm, including rhizome buds germination, shoot sprouting, and young culm development. Meanwhile, samples also included the genets or mother bamboo system (e.g., leave, culm, rhizome) and ramets (e.g., rhizome buds, new bamboo shoots). The weight of each sample was approximately 1–2 g, and three duplicate samples were collected. The samples were quickly collected and stored in liquid nitrogen. After being transported to the laboratory, all samples were stored in a −80 °C refrigerator for subsequent hormone content determination.

### 4.2. Hormone Analysis

An enzyme-linked immunosorbent assay (ELISA) kit (China Agricultural University, Beijing, China) [17,18,19] was used to determine the content of four endogenous hormones with fresh weight: IAA, GA, ABA, and CTK. Each sample was measured three times, repeatedly.

The specific extraction procedures were as follows: (1) weighed 0.2 g of freeze-dried and ground sample, added 5 mL of extraction solution, shook well, and placed the mixture for 4 h at 4 °C according to the instructions of the ELISA kit manufacturer; (2) centrifuged the mixed extraction solution at 3500 rpm/min for 8 min at 4 °C and collected the supernatant with a pipette; then added 1 mL of extraction solution to the remaining precipitate, shook and extracted at 4 °C again, and collected the supernatant again; (3) passed supernatant through a C-18 solid-phase extraction column, dried the sample obtained after passing through the column, and then diluted it to 5 mL with sample diluent from the ELISA kit, then added the standard substance and placed the mixture for 0.5 h at 37 °C; (4) after color development, measured the OD values of the standard substance and each sample at 490 nm with an ELISA spectrophotometer; (5) fitted the standard curve of hormones and calculated the hormone content in each sample using a logit curve based on the measured values.

### 4.3. Seasonal Division

Daweishan National Nature Reserve is located on the southern edge of the subtropical zone. According to local climate characteristics and agricultural habits, the four seasons were divided into: spring (February, March, and April); summer (May, June, and July); autumn (August, September, and October); and winter (November, December, and January).

### 4.4. Data Processing

Data were pre-processed using Microsoft Excel 2016 (Microsoft Corporation, Redmond, WA, USA) and analyzed using SPSS software (v. 20.0; IBM Corporation, Armonk, NY, USA). One-way analysis of variance and Duncan’s test were used to compare the mass fractions and ratios of IAA, ABA, GA, and CTK among developmental periods and bamboo components. Diagrams were visualized using the software Origin 2022 (Origin Lab. Corporation, Northampton, MA, USA).

## 5. Conclusions

The attribute of producing shoots all year round under natural conditions makes *C. pingbianense* different from woody bamboos. It is also interesting that the emerging bamboo shoot amount is not subject to the increasing precipitation and soil temperature in the habitat of *C. pingbianense*. The above phenological phenomena suggest that endogenous hormones may be the dominant factor in bamboo shoot sprouting in this species. Among four examined endogenous hormones in seven structural components, ABA had the highest mass fraction, followed by IAA, CTK, and GA. The bamboo shoot amount is significantly correlated with IAA content and ratios of IAA/ABA, (IAA + CTK + GA)/ABA, and CTK/IAA in the underground components. In particular, the contents of IAA and ABA in rhizome buds maintained a high level throughout the year, suggesting their crucial roles in bamboo shoot sprouting all year round in *C. pingbianense*.

## Figures and Tables

**Table 1:** Shooting phenology in six sample clumps of *C. pingbianense*.

Season/ Month	Spring			Summer			Autumn			Winter			Total
	2	3	4	5	6	7	8	9	10	11	12	1	
Number of emerging shoots	17	18	7	3	1	4	9	15	20	15	14	12	135
Number of degraded shoots	6	5	6	2	1	2	2	3	6	8	5	3	49
Number of grown bamboo	11	13	1	1	0	2	7	12	14	7	9	9	86
Proportion of emerging shoots (amount/%)	42/31.1%			8/5.9%			44/32.6%			41/30.4%			
Proportion of degraded shoots (amount/%)	17/34.7%			5/10.2%			11/22.4%			16/32.7%			
Proportion of grown bamboo (amount/%)	25/29.1%			3/3.5%			33/38.3%			25/29.1%			

## Data Availability

Data are contained within the article.
